# Pre-pectoral Breast Reconstruction: Surgical and Patient-Reported Outcomes of Two-Stages vs Single-Stage Implant-Based Breast Reconstruction

**DOI:** 10.1007/s00266-023-03601-x

**Published:** 2023-08-29

**Authors:** Nicola Zingaretti, Michele Piana, Laura Battellino, Francesca Galvano, Francesco De Francesco, Michele Riccio, Yvonne Beorchia, Luigi Castriotta, Pier Camillo Parodi

**Affiliations:** 1 Department of Medical Area (DAME), Clinic of Plastic and Reconstructive Surgery, Academic Hospital of Udine, University of Udine, Udine, Italy; 2 Accademia del Lipofilling, Research and Training Center in Regenerative Surgery, Jesi, Italy; 3 School of Medicine, University of Udine, Udine, Italy; 4 Department of Oral and Maxillofacial Sciences, Sapienza University of Rome, Rome, Italy; 5 Department of Reconstructive Surgery and Hand Surgery, AOU “Ospedali Riuniti”, Ancona, Italy; 6 Institute of Hygiene and Clinical Epidemiology, University Hospital of Udine, Udine, Italy

**Keywords:** Pre-pectoral breast reconstruction, ADM, Two-stages reconstruction, DTI prosthetic reconstruction, Breast expander

## Abstract

**Background:**

Two-stages pre-pectoral breast reconstruction may confer advantages over direct to implant (DTI) and subpectoral reconstruction in selected patients who have no indication for autologous reconstruction. The primary endpoint of the study was to evaluate and compare the incidence of capsular contracture in the pre-pectoral two-stages technique versus the direct to implant technique. Complications related to the two surgical techniques and patient satisfaction were also evaluated.

**Methods:**

A retrospective review of 45 two stages and 45 Direct-to-implant, DTI patients was completed. Acellular dermal matrix was used in all patients. An evaluation of anthropometric and clinical parameters, surgical procedures and complications was conducted. Minimum follow-up was 12 months after placement of the definitive implant.

**Results:**

There was no statistically significant difference in the rate of capsular contracture in the two groups. Rippling occurred more in DTI reconstruction. In the two-stages reconstruction, lipofilling was applied more often and there was a higher incidence of seroma. Patient satisfaction extrapolated from the Breast Q questionnaire was better for patients submitted to two-stage implant-based breast reconstruction.

**Conclusion:**

Dual-stage pre-pectoral reconstruction with acellular dermal matrix appears to be a good reconstructive solution in patients with relative contraindications for one-stage heterologous reconstruction with definitive prosthesis and no desire for autologous reconstruction.

*Level of Evidence IV* This journal requires that authors assign a level of evidence to each article. For a full description of these Evidence-Based Medicine ratings, please refer to the Table of Contents or the online Instructions to Authors www.springer.com/00266.

## Background

In modern breast reconstruction, the use of minimal demolitive surgical techniques, such as skin sparing and areola-nipple complex sparing (NAC sparing) mastectomy procedures, has greatly improved reconstructive outcomes, bringing the pre-pectoral approach to breast implant placement back into popularity [1–4]. Many surgeons prefer pre-pectoral placement because it allows for utilization of the normal breast space while avoiding animation deformity and reducing postoperative pain by leaving the pectoralis muscle in place [5]. Although pre-pectoral reconstruction is effective, the implant is placed under thin and often poorly vascularized tissue. Therefore, patient selection with careful evaluation of the mastectomy flaps and a correct reconstructive algorithm is of paramount importance. The use of the breast expander is traditionally associated with a two-stage submuscular reconstruction; however, in recent years, it has also begun to be placed in the pre-pectoral site associated with the use of ADM [6–8]. This technique combines the advantages of using the pre-pectoral space with those of skin expansion, thus making it possible to obtain larger volumes in patients who so desire, to expand the skin in the case of large excisions during mastectomy, and not to burden the skin flaps with the weight of the prosthesis. Several studies have demonstrated the effectiveness of one-stage pre-pectoral reconstruction with prosthesis; however, there is still no clear consensus regarding two-stage expander/prosthesis pre-pectoral reconstruction [9]. The primary endpoint of our study was to evaluate the rate of capsular contracture in patients undergoing a two-stage pre-pectoral reconstruction with ADM with a minimum follow-up of one year from the time of definitive implant placement. The complication rates of the individual procedures were also evaluated in comparison with a DTI prosthetic reconstruction, and the histology of the periprosthetic capsule was analyzed. Finally, patient satisfaction was measured using the Breast-Q 2.0 questionnaire.

## Patients and Methods

**Fig. 1:**
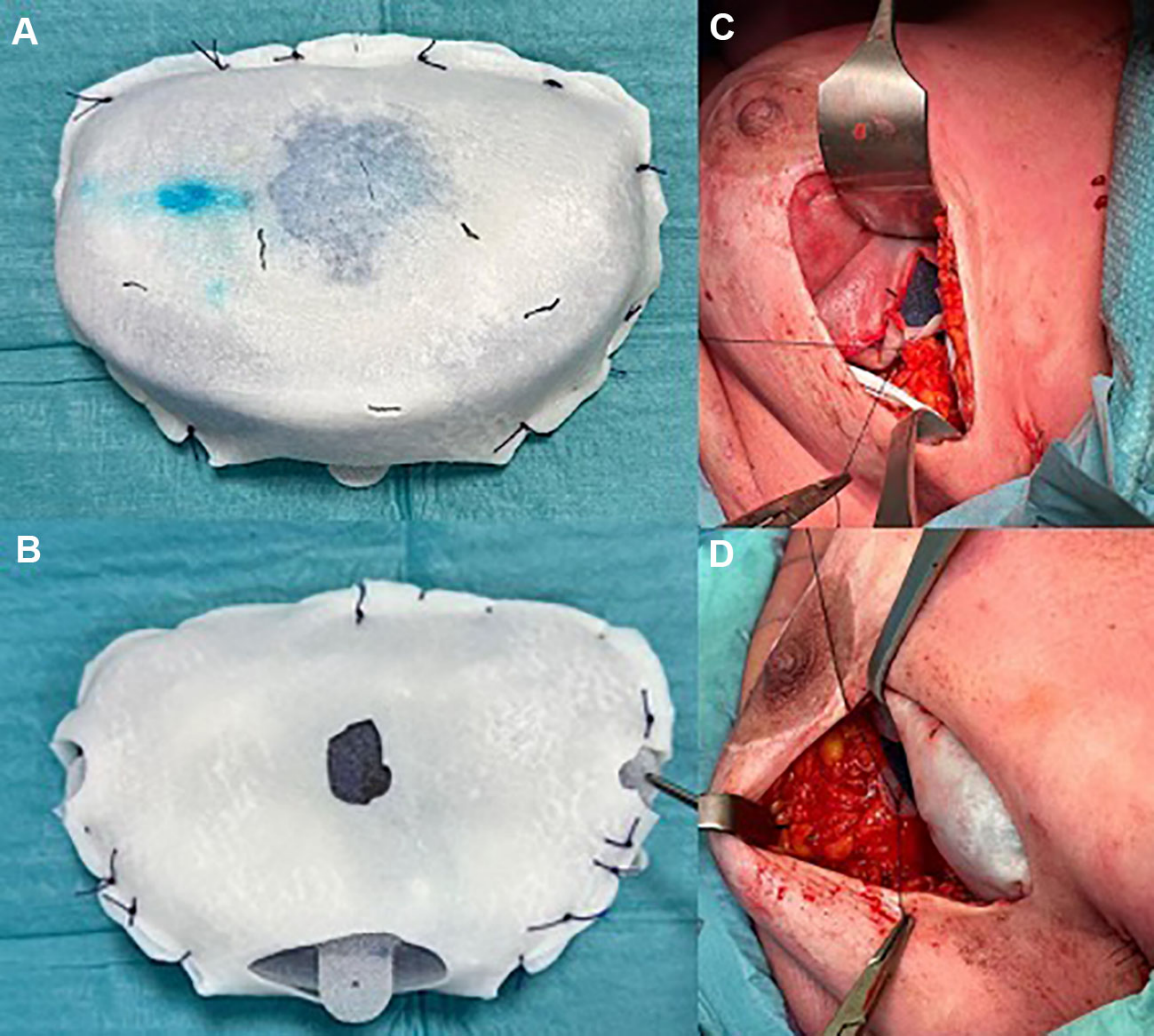
**A**–**B** The expander was fully wrapped with porcine-derived acellular dermal matrix. **C**–**D** The wrapped implant was, then, inserted, and the matrix has been properly fixed in several points (at least three: upper, medially and lower) with resorbable stitches

**Fig. 2:**
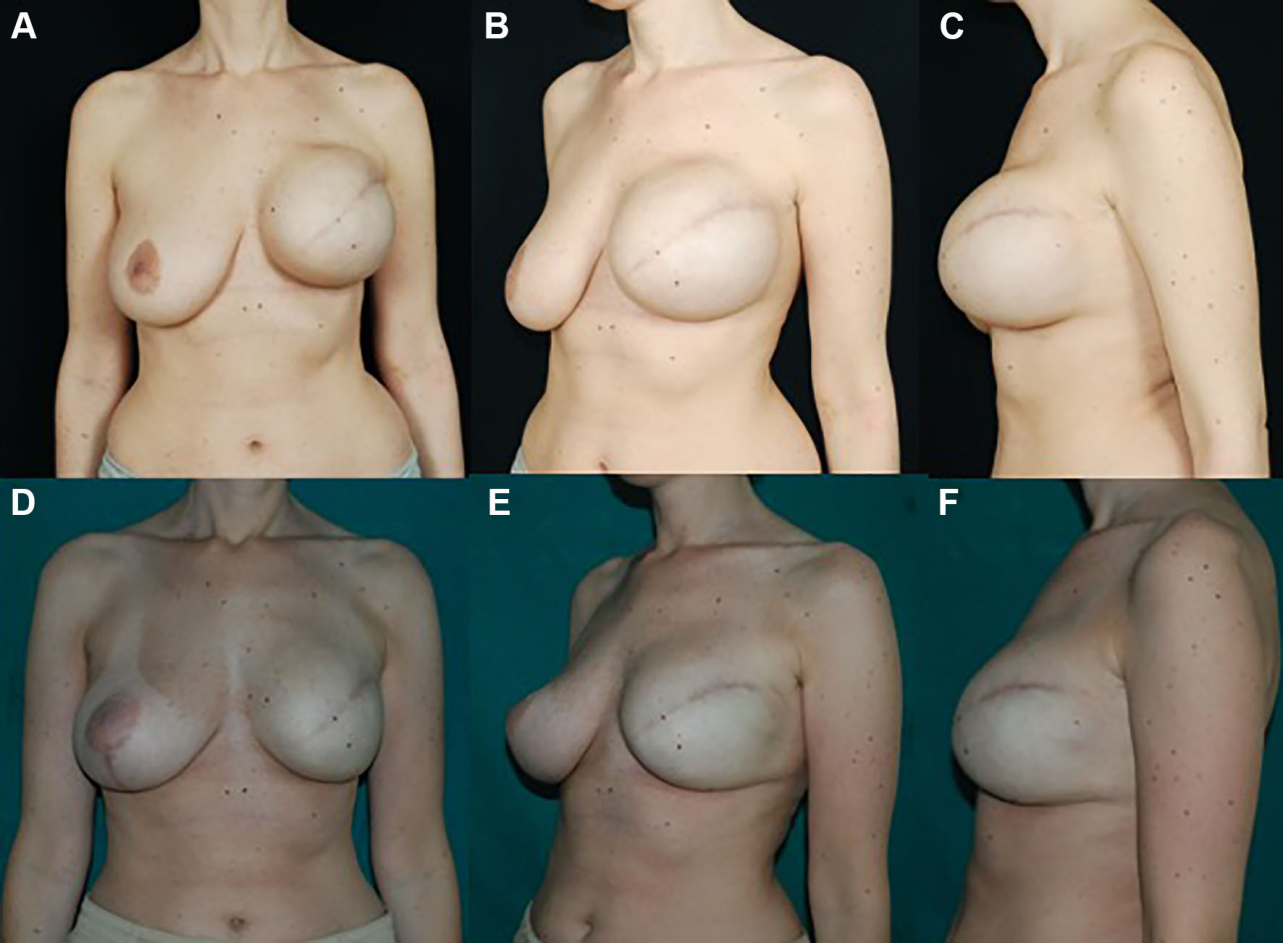
**A**–**C** Patient after 10 months from skin sparing left mastectomy and immediate pre-pectoral reconstruction with tissue expander (Mentor, 450 cc MH) and ADM (Braxon). **D**–**F** Postoperative result after 18 months from the second surgical step of expander/implant exchange procedure. The patient had contralateral mastopexy during the latter surgery

**Fig. 3:**
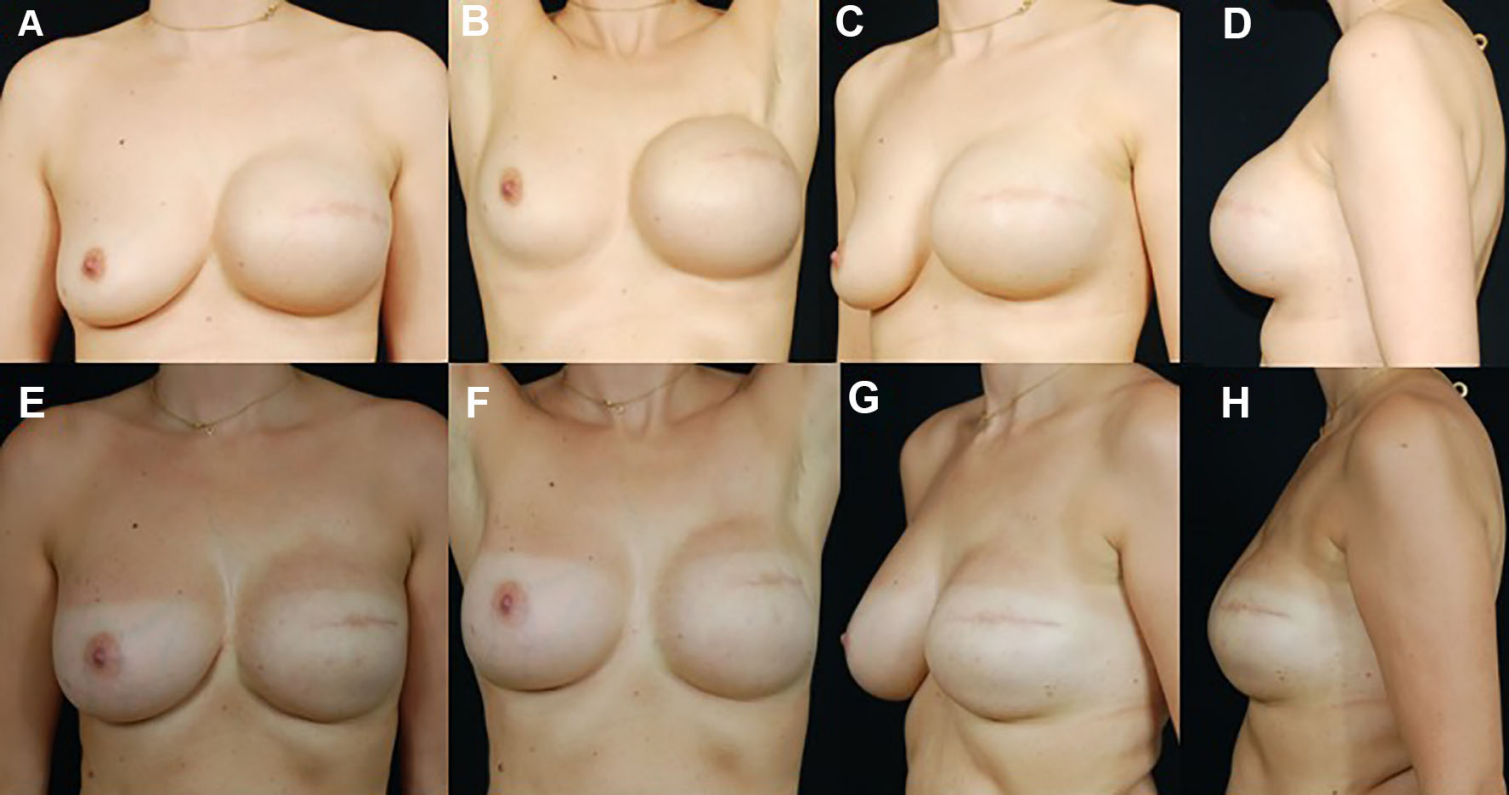
Preoperative pictures (**A**-**D**) of a 47-year-old woman who underwent left skin sparing mastectomy and pre-pectoral expander immediate reconstruction. **E**–**H** postoperative result after 20 months from the second surgical step of expander/implant exchange procedure. The patient had contralateral breast augmentation during the latter surgery

A retrospective observational study was set up analyzing all patients who underwent two-stage breast reconstruction with pre-pectoral expander with ADM and subsequent replacement with implants (Group 1). The control group (Group 2) was set up by including the same number of consecutive patients who underwent pre-pectoral DTI reconstruction with prosthesis with ADM in the same time interval. All patients underwent a pre-operative assessment during which they were informed of the different treatment options (heterologous and autologous). Patients enrolled who underwent postoperative radiotherapy were not scheduled preoperatively; for this reason, a heterologous reconstruction was performed in any case. All patients were operated at the same hospital by the same team consisting of a senior and a junior surgeon. After the mastectomy conducted by the breast surgeon, a clinical evaluation of the mastectomy flaps was performed (indocyanine green). In cases of doubtful or poor tissue viability, a tissue expander was placed retropectorally or reconstruction was postponed, and patients were excluded from the study. If the mastectomy flaps were deemed suitable for immediate single-stage reconstruction, the pre-pectoral pocket was measured, and the patient underwent prosthesis with ADM placement. A textured prostheses (Mentor Corporation, Santa Barbara, Calif., US) were implanted in all patients. The choice of textured prostheses was determined by institutional supply agreements and not by clinical factors. The ADMs used were Braxon (DECOmed s.r.l, Venice, Italy), Surgimend PRS Meshed and Surgimend PRS (Integra LifeScience, Plainsboro, New Jersey, US). In cases where the skin flaps appeared suitable for a pre-pectoral reconstruction, but it was deemed preferable to defer the placement of the definitive implant, the placement of a tissue expander was opted for. The tissue expander was wrapped with ADM that was fixed by resorbable stitches, taking special care to remove any excess matrix not needed to cover the implant. The expander was inflated with saline and methylene blue solution in an amount corresponding to 50 percent of the volume of the breast removed. Suction drains were inserted and kept in place until the 24-hour drainage was less than 30cc. Before implant placement, the breast pocket was washed with disinfectant iodine solution, Gentamicin and Cefazolin. The expander was then inserted into the pre-pectoral pocket and secured to the pectoralis muscle fascia to prevent implant rotation using resorbable sutures (Fig. 1). All patients underwent reconstruction with the same tissue expanders (Mentor Corporation, Santa Barbara, Calif., US). The ADMs used were the same as those used in DTI reconstructions with prostheses. All patients were evaluated regularly every 2 weeks on an outpatient basis, and the expander was progressively inflated with 40–50 cc to the desired volume (the final expansion volume was about 20% greater than the volume of the contralateral breast). All expanders in the study were replaced by anatomical textured breast implants (Mentor Corporation, Santa Barbara, Calif., US) between one and two months after the last filling. If the pinch test was not satisfactory, patients underwent lipofilling either before or after the expander replacement surgery with implants (Figs. 2 and 3). Exclusion criteria were entirely submuscular or dual plane reconstructions, reconstructions with autologous flaps, and delayed reconstructions. The two groups were compared according to the analyzed parameters such as age, BMI, smoking and comorbidities, pre- and postoperative RT and chemotherapy, type and weight of mastectomy, sentinel lymph node and axillary dissection, histology of carcinoma, use of drains, additional surgical procedures, major complications (requiring treatment in the operating theater) and minor complications (managed on an outpatient basis).

Patients were asked to fill in the postoperative BREAST-Q reconstruction questionnaire to evaluate the outcomes. The BREAST-Q reconstruction module has the following scales: satisfaction with breasts, satisfaction with implants, psychosocial well-being, sexual well-being, physical well-being. We administered the questionnaire electronically 1 year after placement of the definitive implant. We compared patient questionnaire scores (group 1 vs group 2) to determine if there was a significant improvement in esthetic outcomes between the groups.

### Statistical Analysis

Descriptive statistics were calculated for continuous variables (means, standard deviations, medians, interquartile ranges, minimum, maximum) and for discrete variables (absolute and relative frequencies). The normality of the distribution of the continuous variables was checked with the Shapiro-Wilk test. Comparisons between categorical variables were carried out with the Chi-square test or Fisher’s exact test. For the comparison of continuous variables in two or more groups, the *t*-test for independent samples, the Wilcoxon–Mann–Whitney test and the Kruskal–Wallis test were used, as appropriate. The correlation between variables was assessed—depending on the distribution—by means of Pearson’s correlation index or Spearman’s rank correlation coefficient. The association of factors with capsular contracture was further evaluated by bivariable logistic analysis, and only those factors that showed a bivariate association with the dependent variable at the significance level *P* < 0.20 were included in the multivariable logistic model. Multicollinearity was assessed with Variance Inflation Factor (VIF), with values above 2.5 indicating considerable collinearity [10]. All tests were 2-sided, and the significance level was set at *P* < 0.05.

The analyses were performed using STATA software (Release 17 StataCorp LP, College Station, TX, USA).

## Results

Patients’ demographics are reported in Table 1.

**Table 1:** Patient demographics, time to revision, follow-up, and presenting clinical signs

	Group 1 (*n* = 45) 1 t	Group 2 (*n* = 45) 2 t	Total (*n* = 90)	*P *value
Age (mean, years)	49.47	52.38	50.92	0.18
Weight (mean, kg)	62.96	64.02	63.49	0.72
BMI (mean, kg/m^2^)	22.71	24.05	23.38	0.11
Overweight (BMI* > *25 kg/m^2^)	7	17	24	0.02
Smoker (%)	10 (22)	10 (22)	20 (22)	1.00
Hypertension (%)	1 (2)	7 (16)	8 (9)	0.77
Diabetes mellitus (%)	0	3 (7)	3 (3)	0.24
Neoadjuvant radiotherapy (%)	5 (11)	1 (2)	6 (7)	0.20
Adjuvant radiotherapy (%)	5 (11)	10 (22)	15 (17)	0.16
Neoadjuvant chemotherapy (%)	4 (9)	6 (13)	10 (11)	0.50
Adjuvant chemotherapy (%)	22 (49)	15 (33)	37 (41)	0.13
*Tumor histotype*				
CaDI (%)	39 (87)	20 (44)	59 (66)	
CaDis (%)	4 (9)	13 (29)	17 (19)	
CaLI (%)	2 (4)	12 (27)	14 (16)	
Mastectomy weight (mean, g)	298.16	357.62	327.89	0.02*
*Type of mastectomy*				
NAC sparing (%)	33 (73)	25 (56)	58 (64)	
Skin sparing (%)	9 (20)	20 (44)	29 (32)	
Skin reducing (%)	3 (7)	0	3 (3)	
Sentinel lymph node biopsy(%)	37 (82)	39 (87)	76 (84)	0.56
Axillary dissection	8 (18)	10 (22)	18 (20)	0.60
*Type of implant*				
CPG 311	6 (13)	2 (4)		
CPG 312	27 (60)	16 (35)		
CPG 313	3 (7)	0		
CPG 321	6 (13)	8 (18)		
CPG 322	2 (4)	10 (22)		
CPG 323	1 (2)	9 (20)		
Implant volume (median, cm^3^)	268,69	408.11	353.4	0.04
*ADM*				
Braxon	24 (53)	22 (49)	46 (51)	
Surgimend PRS meshed	17 (38)	19 (42)	36 (40)	
Surgimend PRS	4 (9)	4 (9)	8 (9)	
Contralateral symmetrization	6 (13)	11 (24)	17 (19)	
*Drainage*				
One	22 (49)	12 (27)	34 (38)	0.18
Two	23 (51)	33 (74)	56 (62)	0.03*
Days of drain permanence (mean, days)	9.67	12.98	11.32	0.001*
Length of follow-up (range, months)	15–43	14–38		

* *P* < 0.05

1 t: One-stage immediate breast reconstruction

2 t: Two-stage implant-based breast reconstruction

A total of 45 patients with a mean age of 52.3 years (range 29–78 years) and mean body mass index of 24 kg/m^2^ (range 18–35 kg/m^2^), respectively, underwent a total of 45 pre-pectoral breast reconstructions with tissue expander with ADM. The most commonly ADM used were Braxon (22 patients), followed by Surgimend PRS Meshed (19 patients) and Surgimend PRS (4 patients). In the control group, a total of 45 patients with a mean age of 49.5 years (range 23–71 years) and mean body mass index of 22.7 kg/m^2^ (range 18–33.8 kg/m^2^), respectively, underwent a total of 45 pre-pectoral breast reconstruction with implant with ADM. Also, in this case the most frequently used ADM was Braxon (24 patients) followed by Surgimend PRS Meshed (17 patients) and Surgimend PRS (4 patients). Taking into consideration the risk factors of the patients reconstructed with an expander (Group 1), 3 patients (6.7%) had diabetes mellitus, 7 operations (15.6%) were performed on high blood pressure patients and 10 patients (22.2%) were smokers. None of the patients reconstructed in single-time surgery were diabetic, only 1 patient (2.2%) had high blood pressure, and 10 (22.2%) were smokers. In the two-stage reconstruction (Group 2), in 1 case (2.2%) the patient received neoadjuvant RT prior to expander placement, in 10 cases (22.2%) adjuvant radiotherapy; in 6 cases (13.3%), the patients received neoadjuvant chemotherapy and in 15 received (33.3%) adjuvant chemotherapy; in DTI reconstruction, 5 patients (11.1%) received neoadjuvant radiotherapy prior to implant placement and 5 (11.1%) received adjuvant radiotherapy; 4 patients (8.9%) received neoadjuvant chemotherapy and 22 (48.9%) received adjuvant chemotherapy. Among the patients reconstructed in two stages (Group 2), 39 (86.7%) underwent sentinel lymph node biopsy, 10 (22.2%) underwent axillary dissection. The most represented tumor histotype was infiltrating ductal carcinoma in 24 cases (44.4%), followed by ductal carcinoma in situ 13 (28.9%). The mean mastectomy weight was 357 grams (range 160–787 grams). Eleven patients (24.4%) underwent contralateral symmetrization. In 12 breasts (26.7%) was placed only one drain, in 33 breasts (73.3%) two drains were placed, and all drains were kept in place for an average of 13 days (range 6–20 days).

The most frequent complication (Table 2) after expander placement (Group 2) was seroma, minor in 22 cases (48.9%), followed by superficial necrosis of skin flaps in 10 cases (22.2%). Twenty-one patients (46.7%) underwent lipofilling before expander replacement, ten patients (22.2%) after implant placement. The average implant volume was 410 cc (range 145–685cc).

**Table 2:** Number of postoperative complications

	Group 1 (*n* = 45) 1 t	Group 2 (*n* = 45) 2t	Total (*n* = 90)	*P* value
Seroma minor (%)	12 (27)	22 (49)	34 (38)	0.03*
Seroma major (%)	1 (2)	0	1	1
Haematoma (%)	7(16)	4 (9)	11 (12)	0.33
Infection (%)	1 (2)	2 (4)	3 (3)	1
Superficial Necrosis of skin flap (%)	6 (13)	10 (22)	16 (18)	0.27
Capsular contracture (grade III-IV)	6 (13)	2 (4)	8 (9)	0.27
Rotation/DIslocation	1 (2)	0	1 (1)	1
Rippling/Wrinkling	17 (38)	8 (18)	25 (28)	0.03*
Lipofilling before expander replacement		21 (47%)		
Lipofilling after implant placement	9 (20)	10 (22)	19 (21)	0.8

* *P* < 0.05

1 t: one-stage immediate breast reconstruction

2 t: two-stage implant-based breast reconstruction

In the DTI group (Group 1), thirty-seven patients (82.2%) underwent sentinel lymph node biopsy, and 8 patients (17.8%) underwent axillary dissection. The most represented tumor histotype was infiltrating ductal carcinoma in 39 cases (86.7%) followed by ductal carcinoma in situ in 4 (8.9%). The mean mastectomy weight was 298 grams (range 90–768 grams). Six patients (13.3%) underwent contralateral symmetrization. In 22 breasts (48.9%), only one drain was placed; in twenty-three cases, (51.1%), two drains were placed; all drains were kept in place on average for 10 days (range 3–21 days). The most frequent complication after reconstruction was minor seroma in twelve breasts (26.7%), followed by haematoma in seven breasts (15.6%). Nine patients (20.0%) underwent lipofilling after implant placement (group 2). The average implant volume was 298 cc (range 120–585cc).

The results of univariable and multivariable logistic regression analyses are presented in Table 3. In univariable logistic regression, four independent variables were found to meet the criteria to be included in the multivariate model (*P* < 0.20): age, type of surgery (two-stage vs DTI reconstruction), volume, and neoadjuvant chemotherapy. Findings from multivariable model, which satisfies the hypothesis of absence of considerable multicollinearity, showed that age was indirectly associated with capsular contracture (OR = 0.90. 95%CI = 0.82–0.99), whereas neoadjuvant chemotherapy appeared to increase the likelihood of developing this complication (OR = 13.32, 95%CI = 1.19–148.89). Women with a larger volume also seem to have a lower risk of capsular contracture, although statistical significance was not reached (OR = 0.99, 95%CI = 0.98–1.00). Two-stage surgery, on the other hand, does not appear to significantly reduce this complication compared with DTI reconstruction (OR = 0.38, 95%CI = 0.05–3.04).

**Table 3:** Univariable and multivariable logistic regression analysis of association between capsular contracture and related factors

Univariate analysis					
	OR	SE	*z*	95% CI	*P value*
Age	0.88	0.04	− 2.79	(0.81–0.96)	0.005**
BMI	0.91	0.11	− 0.77	(0.72–1.15)	0.444
Overweight	0.91	0.78	− 0.11	(0.17–4.84)	0.911
*Type of surgery*					
One-stage reconstruction (ref)					
Two-stage reconstruction	0.3	0.26	− 1,41	(0.06–-1.59)	0.157
Mastectomy weight (g)	1	0	− 0.99	(0.99–1.00)	0.322
Volume (cc)	0.99	0	− 1.91	(0.99–1.00)	0.056
Postoperative radiotherapy	3.5	2.78	1.58	(0.74–16.6)	0.115
Neoadjuvant chemotherapy	6.43	5.34	2.24	(1.26–32.73)	0.025*
Adjuvant chemotherapy	2.6	1.99	1.25	(0.58–11.65)	0.211
Smoking	1.19	1.02	0.2	(0.22–6.38)	0.843
*Multivariable analysis*					
	OR	SE	*z*	95% CI	*P value*
Age	0.9	0.04	− 2.18	(0.82-0.99)	0.029*
*Type of surgery*					
One-stage reconstruction (ref)					
Two-stage reconstruction	0.38	0.4	− 0.91	(0.05–3.04)	0.363
Volume (cc)	0.99	0.01	− 1.72	(0.98–1.00)	0.086
Neoadjuvant chemotherapy	13.32	16.41	2.1	(1.19-148.89)	0.036*

< 0.10; * < 0.05; ** < 0.01; *** < 0.001

OR, Odds Ratio; SE, Standard Error; CI, Confidence Interval; BMI, Body Mass Index

Forty patients of group 1 and thirty-eight of group 2 completed BREAST-Q surveys, with a response rate of 89% for group 1 and 84% for group 2. The answers of the patients to the BREAST-Q are shown in Table 4. After further analysis of the questionnaire, we observed that patients in group 2 obtained significantly better postoperative results than patients from group 1 (control) regarding the following items: the reconstructed breast softness, symmetry (breasts of equal size relative to the other), reconstructed breast look and touch, amount of implant rippling perceived by the patients, and physical well-being about chest and upper body.

**Table 4:** BREAST-Q postoperative module applied in group 1 and 2 one year after last procedure

Question	Post (group 1)	Post (Group 2)	P (1 year)
No (%)	40 (89)	38 (84)	–
*Satisfaction with breasts*			
How you look in the mirror clothed?	25 (63)	27 (71)	0.34
The shape of your reconstructed breast(s) when you are wearing a bra?	29 (72)	30 (78)	0.54
How normal you feel in your clothes?	31 (77)	31 (81)	0.66
The size of your reconstructed breast(s)?	34 (85)	34 (89)	0.63
Being able to wear clothing that is more fitted?	30 (74)	30 (80)	0.49
How your breasts are lined up in relation to each other?	29 (72)	34 (90)	0.01*
How comfortably your bras fit?	31 (77)	33 (86)	0.24
The softness of your reconstructed breast(s)?	21 (53)	32 (84)	0.0001*
How equal in size your breasts are to each other?	32 (80)	34 (90)	0.13
How natural your reconstructed breast(s) looks?	26 (64)	27 (70)	0.04
How naturally your reconstructed breast(s) sits/hangs?	20 (50)	33 (88)	0.0001*
How your reconstructed breast(s) feels to touch?	22 (54)	21 (56)	0.78
How much your reconstructed breast(s) feel like a natural part of your body?	22 (54)	21 (56)	0.78
How closely matched (similar) your breasts are to each other?	22 (54)	23 (60)	0.72
How you look in the mirror unclothed?	18 (44)	20 (54)	0.30
*Satisfaction with implants*			
The amount of rippling (wrinkling) of your implant(s) that you can see?	26 (65)	12 (32)	0.0001*
The amount of rippling (wrinkling) of your implant(s) that you can feel?	26 (65)	12 (32)	0.0001*
*Psychosocial well-being*			
Confident in a social setting?	33 (82)	32 (84)	0.82
Emotionally able to do the things that you want to do?	31 (77)	29 (75)	0.77
Emotionally healthy?	33 (82)	32 (84)	0.79
Of equal worth to other women?	32 (80)	29 (77)	0.56
Self-confident?	34 (86)	32 (83)	0.56
Feminine in your clothes?	36 (89)	34 (90)	0.84
Accepting of your body?	35 (88)	34 (90)	0.67
Normal?	35 (88)	33 (86)	0.63
Like other women?	36 (89)	34 (90)	0.84
Attractive?	29 (72)	26 (68)	0.48
*Sexual well-being*			
Sexually attractive in your clothes?	30 (74)	27 (71)	0.81
Comfortable/at ease during sexual activity?	25 (63)	23 (60)	0.77
Confident sexually?	29 (72)	27 (70)	0.77
Satisfied with your sex-life?	29 (73)	28 (75)	0.84
Confident sexually about how your breast area looks when unclothed?	22 (54)	21 (56)	0.78
Sexually attractive when unclothed?	18 (44)	21 (55)	0.16
*Physical well-being*			
Neck pain?	16 (39)	13 (35)	0.69
Upper back pain?	15 (38)	13 (35)	0.80
Shoulder pain?	15 (38)	14 (37)	0.95
Arm pain?	12 (29)	10 (27)	0.76
Rib pain?	18 (45)	12 (32)	0.10
Pain in the muscles of your chest?	26 (65)	12 (32)	0.0001*
Difficulty lifting or moving your arms?	12 (29)	10 (27)	0.76
Difficulty sleeping because of discomfort in your breast area?	18 (44)	12 (32)	0.13
Tightness in your breast area?	24 (59)	13 (35)	0.005*
Pulling in your breast area?	23 (57)	13 (35)	0.008*
Nagging feeling in your breast area?	23 (57)	13 (35)	0.008*
Tenderness in your breast area?	18 (44)	18 (47)	0.80
Sharp pains in your breast area?	26 (65)	12 (31)	0.0001*
Shooting pains in your breast area?	29 (72)	17 (46)	0.0007*
Aching feeling in your breast area?	28 (71)	13 (35)	0.0001*
Throbbing feeling in your breast area?	29 (72)	16 (41)	0.0001*

* *P* < 0.01 was accepted as the level of statistical significance

Raw score ≥ 4

## Discussion

Breast carcinoma is the most widespread non-skin malignancy in the female population, and it also is the most common cause of death due to malignancy [1–4]. Alongside the primary objective of oncological radicality, breast reconstruction treatment has developed over the years, aimed at restoring the integrity of the breast, a pivotal element in determining female identity, in aesthetic as well as functional terms [11, 12].

In the past, pre-pectoral breast reconstruction after mastectomy without the use of ADM showed a high rate of capsular contracture, implant exposure and subsequent submuscular conversion [13, 14]. However, the advent of ADM has made pre-pectoral reconstruction a safe technique when applied in appropriately selected patients, showing relevant aesthetic results. Many authors cite a number of reasons for their use, including better control of the prosthetic pocket resulting in superior aesthetic results, less expansion required to achieve the final volume, better definition of the inframammary fold, and a reduced risk of capsular contracture evolution. Furthermore, this type of reconstruction leaves the pectoral muscle unaltered, avoiding animation deformity and reducing postoperative pain [13, 14].

The primary endpoint of the study was the evaluation of the incidence of capsular contracture in these two surgical techniques. In 2 patients (4.4%), undergoing two-stage reconstruction and in 6 patients (13.3%), undergoing DTI reconstruction a significant degree of capsular contracture (Baker III-IV) were found, while in the rest of the sample there was no evidence of contracture, with a follow-up of at least 12 months after placement of the definitive implant. The difference between the two groups of patients did not shows statistical significance (*p = *0.27). These results appear to be similar to those found in the literature; however, in order to further validate the quality of the technique, it will be necessary to evaluate the patients after a longer follow-up [14, 15]. The data from our study thus confirms the evidence in the literature, which describes the usefulness of ADM in preventing the development of capsular contracture [16]. Liu et al. in a 2020 meta-analysis compared the incidence of capsular contracture in ADM versus non-ADM reconstructions, with the following results: reconstructions with ADM had an incidence of capsular contracture of 2.4%, compared to non-ADM where the incidence was 6–18% [17]. Capsular contracture consists of the retraction of the capsule surrounding the prosthesis, which is formed by the body’s natural reaction to a foreign body; the local inflammatory response is considered the primary etiopathogenetic mechanism, protracted inflammation leads to fibrosis, which in time leads to the development of tension and shrinkage of the capsule until capsular contracture is determined [4, 18]. And it is precisely by limiting this inflammatory process that ADM are advantageous in reducing the incidence of capsular contracture. Salibian et al analyzed the different incidence of complications between two cohorts, which underwent pre-pectoral breast reconstruction with expander and ADM (Alloderm and Flex HD) and without matrix, respectively [19]. The results of this study demonstrated a low and comparable rate of complications between two cohorts. On the other hand, the positive impact of the introduction of ADMs in the reduction in capsular contracture is remarkable, although this study also needs a longer follow-up to confirm the results obtained.

The effects of radiotherapy are especially evident in submuscular or dual plane reconstructions, and the triggering factor is the fibrosis that is created in the muscle tissue, which leads to contraction and consequent dislocation of the implant in a more cranial position [20]. In more severe cases, the contracture of the capsule becomes painful, making it unbearable for the patient to keep the implant in place. Placing the implant in a pre-pectoral position, sparing the pectoral muscle incision, greatly reduces the rate of capsular contracture. In addition, radiotherapy has the side effect of reducing skin elasticity; therefore, the placement of a tissue expander makes it possible to counteract this mechanism and allow the prosthesis to position itself more naturally by adapting to the skin envelope [21, 22]. The use of the pre-pectoral expander with ADM also allows patients who would normally have required an autologous reconstruction to receive a heterologous reconstruction, leaving room for a definitive reconstructive choice at a later date. In fact, these patients will be able, if the condition of the tissues after skin expansion allows it, to receive a pre-pectoral reconstruction with a definitive prosthesis; on the other hand, if it is preferable to proceed with an autologous reconstruction, the expander will have left an ideal space for a pre-pectoral pocket suitable to accommodate a buried flap while maintaining the pectoral muscle unchanged and providing a superior aesthetic outcome.

The second objective of the study was to analyze the incidence of major postoperative complications in the two groups at the end of the first surgical time of tissue expander placement or following DTI reconstruction with prosthesis. Major complications were considered to be those that required revision surgery in the operating room, while minor complications were those that were resolved spontaneously or by outpatient surgery only [23].

While the contribution of ADM in reducing capsular contracture is well established in the literature, the same cannot be said for complications such as seroma, haematoma and infection; in fact, there is conflicting data and expert opinion in the literature on this subject [24, 25]. Despite the known usefulness of ADM, there are also some critical issues associated with their use, among them a potential increase in the likelihood of postoperative complications, mainly seroma and infection, with results appearing disparate and not always concordant in the literature. Therefore, ADMs are expensive and if not used appropriately may present difficulties in integrating into the host tissue, with the aforementioned complications [26].

Seroma is the most frequent postoperative complication after mastectomy: the seroma formation in pre-pectoral reconstruction with implant and ADM ranging from 5% up to 61% [27, 28]. In our study, the incidence of seroma was higher than in the literature, with a value of 48.9%; the limitation of this value is, however, related to a failure to distinguish according to the extent of the seroma: all seromas found whenever even the slightest presence of seroma was detected during the inflation of the expander were taken into account. For many authors, in fact, seroma that develops within the first month and resolves spontaneously or by ambulatory puncture is not considered as a true complication, but as a normal surgical consequence [29]. In the treatment of seroma, the use of drains is of great importance; many authors recommend the use of two drains especially in large breasts and to maintain them for at least two weeks. In our sample, the drains were kept in place for 13 days in two-stage reconstruction and 10 days in single-stage reconstruction (*p = *0.03). The pre-pectoral use of tissue expander together with ADM has the advantage of being able to aspirate any seroma on an outpatient basis without the need for ultrasonography and eliminating the risk of damage to a definitive implant [30]. The aspirated fluid can be subjected to a culture examination so that any infection can be treated promptly with antibiotic therapy without having to resort to removing the implant. Although Momeni et al. reported that a positive culture examination does not always have to be followed by antibiotic therapy, it could be an important information that can be set up in a targeted manner when there is an appearance of clinical signs of infection [26].

In our study, the incidence of infection and partial necrosis of the mastectomy flaps is also close to the data in the literature for both groups, respectively, 0–7% for infection and 0–21 % for mastectomy flap necrosis [31]. Our results showed no statistically significant correlation between the type of operation and the development of infection (*p = *1.00) and mastectomy flap necrosis (*p = *0.27). However, it should be considered that using the partially filled expander intraoperatively gives the opportunity to place less weight and skin tension on the mastectomy flaps in the immediate postoperative period reducing the risk of skin necrosis [32]. It is then possible to adapt the reconstruction in the second surgical time according to the new anatomical framework and the patient’s expectations. The subsequent expansion thus also allows for the use of larger implants that would have been contraindicated in a DTI reconstruction with prosthesis and ADM [33].

Our results show that volume of the definitive implant was significantly greater (*p = *0.04) in patients who received a two-stage reconstruction (with an average of 410 cc) compared to patients undergoing DTI reconstruction (average 300 cc). The breast expander also makes it possible to achieve larger volumes in patients with small breasts who want an increase in breast volume by undergoing breast augmentation surgery on the contralateral breast. Analyzing our sample, we detect a statistically significant difference in mastectomy weight with an average of 298 grams in patients who underwent single-stage reconstruction and 357 grams in patients who underwent two-stage reconstruction (*p = *0.02). This result suggests that with a larger starting breast volume, the final reconstruction was postponed, allowing tissue expansion and pre-pectoral pocket adaptation without the risk of skin necrosis with implant exposure.

When breast expander is overfilled, it can compromise the perfusion of the mastectomy flaps, resulting in complications [19]. The quality of the mastectomy flaps is also a strong predictor of success or failure of the operation, altering the ability of the ADM to integrate with the surrounding tissues; so where the viability of the skin envelope is compromised, it would be better to opt for submuscular reconstruction or delayed reconstruction. Closely related to breast volume is the patients’ BMI; in fact, in our study, 17 patients reconstructed with an expander were overweight (BMI* > *25), while only 7 were overweight among those reconstructed with implants and ADM (*p = *0.02).

As reported in the literature, a high BMI, diabetes mellitus, smoking and hypertension can cause damage to the microcirculation, facilitating phenomena of tissue suffering and necrosis, and increasing the risk of infections following surgical procedures, as well as slowing down the reparative processes [34]. Furthermore, patients with BMI* > *25 often present a large and ptotic breasts. In this case, the use of pre-pectoral expander seems to be an ideal choice because the aspiration of postoperative seroma (more frequent in overweight patient) could be easier compared to the use of definitive implant; furthermore, could allow for a natural breast ptosis (after placement of the definitive implant) and the possibility of obtaining a satisfactory final volume without the need to perform the contralateral breast symmetrization.

Haematoma has a multifactorial etiology in that both factors related to the patient (blood pressure, anticoagulant therapy, etc.) and to the surgical procedure (haemostasis, compression devices, etc.); all these factors are difficult to assess and sometimes unpredictable. The values found in our cohort of patients are closer to those found in the literature (from 2 to 20%) [25]. Our data showed that the incidence of haematoma was 8.9% in patients who received a two-stage reconstruction and 15.6% in patients who received a DTI reconstruction; again, the statistical analysis did not show a statistically significant correlation with the type of operation (*p = *0.06). The higher incidence of haematoma in patients reconstructed with prosthesis, and ADM could be related to the greater weight of the definitive implant: the ADM that surrounds the prosthesis is fixed to the pectoralis major muscle and excessive traction on the sutures could cause bleeding.

Rippling/wrinkling is a typical complication of pre-pectoral reconstruction, especially in thin patients; the main causes of this complication are the placement of an oversized prosthesis and/or a thin mastectomy flap [35]. In our case series, we found a higher incidence of rippling in patients undergoing DTI (17 cases) than in patients reconstructed in two-stage surgery (8 cases) (*p = *0.03). We believe that this finding is due to several factors: first of all to the fact that the patients in our sample reconstructed in single-stage surgery have a lower BMI with a higher number of overweight patients among those reconstructed with expander. Furthermore, lipofilling procedures were more frequently performed in patients submitted to two-stage surgery. Finally, let us consider how a two-stage reconstruction allows the skin envelope to adapt and recover its trophism after mastectomy surgery and possible radiotherapy; it is known, in fact, how the use of ionizing radiation creates an alteration in tissue trophism and increases its fibrosis, compromising the final reconstructive results [36]. In our case series, lipofilling was performed during the expansion phase to avoid excessive skin thinning and reduce the risk of implant exposure. In some patients, it was performed at the same time as the replacement of the expander with a prosthesis to give more support to the implant and to allow the placement of a smaller prosthesis increasing the skin thickness with consequent reduction in the risk of capsular contracture and improvement of the aesthetic outcome [2, 37]. Finally, in some patients we infiltrated adipose tissue in the follow-up phase to correct minor shape defects or to reduce rippling. Among patients reconstructed in two surgical stages, 21 underwent lipofilling with the expander in place or during replacement and 10 after placement of the definitive prosthesis. On the other hand, among patients reconstructed with prosthesis and ADM, this procedure was used 9 times (*p = *0.01). This difference is due to the fact that DTI reconstruction is reserved for selected patients, who present a good pinch test of mastectomy flap in a preoperative phase and do not present significant risk factors. Otherwise, two-stage reconstruction with an expander is offered to patients who are not ideal candidates for DTI reconstruction but can still benefit from preoperative reconstruction. In this case, fat grafting allows us to correct the characteristics of a non-ideal or radio-treated skin envelope [2].

**Fig. 4:**
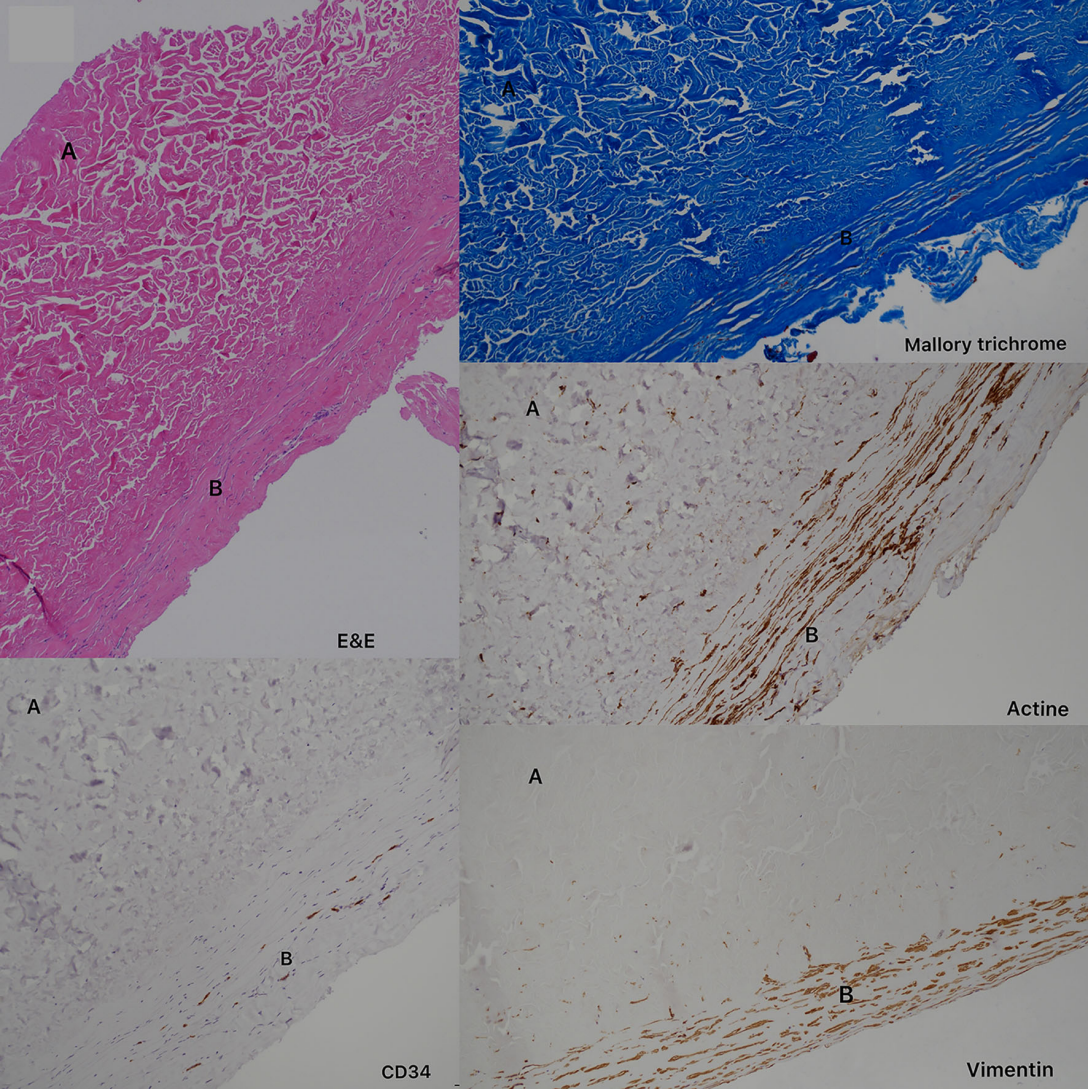
Microscopically, most of the tissue was presented by dense fibrous tissue (EE and Mallory’s trichrome stain). The dense fibrous tissue of the specimen had two separate areas. One area A [up left] had disorganized haphazardly oriented collagen fibers; it was acellular and avascular, as decellularized matrix. The other area B [down right] comprised collagen fibers arranged in thick bundles and oriented in a similar direction. In area B, myofibroblasts (vimentin and Actin positive cells) were present between collagen bundles, and there were many small vessels (CD34 positive). The pattern of this area was highly suggestive of mature dense connective tissue like matrix that had undergone tissue remodeling and revascularization

**Fig. 5:**
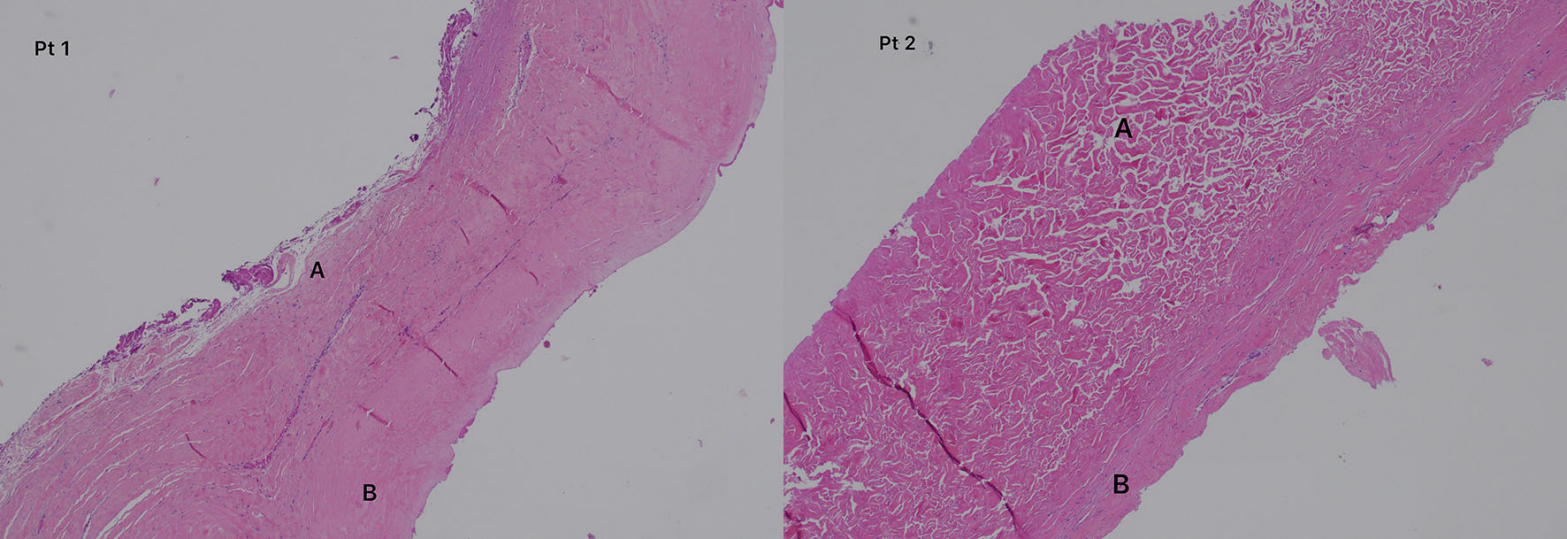
Microscopically the capsule of two different patient had similar organization (on the left Group 1 and on the right Group 2). Area A shows disorganized pink collagen fibers. Note the absence of cells or vessels—an acellular area—consistent with acellular dermal matrix. Area B shows features of an organized dermis with oriented pink collagen fibers, rare fibroblasts, and neovascularization. These features are consistent with reorganized dermal matrix

All of our patients underwent capsular sampling during the expander replacement surgery with the prosthesis, and an histological analysis was performed; no significant differences were found in our case series compared to ADM placed with prosthesis (Figs. 4 and 5), and confirmed was found in the literature [38].

The last objective of the study was to assess the subjective satisfaction of individual patients by administering the BREAST-Q questionnaire [39, 40]. Our data reveal a medium to high degree of satisfaction with the reconstruction performed (Table 3). The topics addressed in the questionnaire concerned psychosocial and sexual well-being and postoperative satisfaction with one’s breasts. The results obtained are encouraging and show substantial satisfaction even in patients who had a less than ideal starting condition for heterologous reconstruction.

Our pilot study confirmed that the use of ADM in two-stage pre-pectoral breast reconstruction is a good option (in terms of satisfaction and complication rate) for patients with no indication for DTI reconstruction. Although the use of ADM and a dual-stage reconstruction involve a more surgical procedure and higher costs, when we consider the repercussions of failure of a DTI reconstruction, we realize that this surgical technique may be the most effective and advantageous choice in patients who are not candidates for immediate permanent implant placement.

### Limitations of the Study

The main limitations of the study were the sample size, the use of only textured anatomical prostheses belonging to a single brand, the short follow-up, and the retrospective study design [41]. Although the use of ADM has already been described in the literature as a favorable factor in the reduction in capsular contracture, the underlying mechanisms are still under investigation [42]. Then, consider that only three types of dermal matrix from two different brands were used in the study. Furthermore, the patients were not randomly distributed but were placed into the two groups based on specific characteristics. We believe that further prospective studies with larger case series and longer follow-ups are needed to confirm the efficacy of this reconstructive approach as we found it, or even its superiority over conventional methods. An analysis of the costs associated with the use of ADM and the need for a ‘double surgery’ would appear to be useful to confirm the applicability of this procedure, although the characteristics of each individual patient must be considered [43].

Finally, it should be remembered that the results obtained refer to a selected group of patients with well-defined characteristics and may therefore not be applicable to a more heterogeneous group of patients.

## Conclusion

Two-stage breast reconstruction with expander and ADM is a recently introduced technique, but one that is gaining ground due to the advantages that the use of biological matrices has on the outcomes of mastectomy patients associated with the use of an established technique such as skin expansion. Our pilot study confirmed how the use of ADM allows for good reconstructive results in patients who would have had contraindications to reconstruction in the past. In terms of periprosthetic capsular contracture, the main outcome of the study, we noticed no significant differences compared to patients undergoing DTI reconstruction. In relation to minor complications, the complications seem to be superimposable in the one-stage reconstruction group (although there is an increased rate of seromas). Therefore, dual-stage pre-pectoral reconstruction with ADM appears to be a good reconstructive solution in patients with relative contraindications for one-stage heterologous reconstruction with definitive prosthesis and no desire for autologous reconstruction. Finally, the analysis of the patients’ postoperative satisfaction showed a medium-high degree of satisfaction with the results obtained.
